# Glutaminolysis and peripheral CD4^+^ T cell differentiation: from mechanism to intervention strategy

**DOI:** 10.3389/fimmu.2023.1221530

**Published:** 2023-07-21

**Authors:** Tong Liu, Shaohua Ren, Chenglu Sun, Pengyu Zhao, Hao Wang

**Affiliations:** ^1^ Department of General Surgery, Tianjin Medical University General Hospital, Tianjin, China; ^2^ Tianjin General Surgery Institute, Tianjin, China

**Keywords:** CD4 + T cells, glutaminolysis, T cell differentiation, immunometabolism, intervention strategy

## Abstract

To maintain the body’s regular immune system, CD4^+^ T cell homeostasis is crucial, particularly T helper (Th1, Th17) cells and T regulatory (Treg) cells. Abnormally differentiated peripheral CD4^+^ T cells are responsible for the occurrence and development of numerous diseases, including autoimmune diseases, transplantation rejection, and irritability. Searching for an effective interventional approach to control this abnormal differentiation is therefore especially important. As immunometabolism progressed, the inherent metabolic factors underlying the immune cell differentiation have gradually come to light. Mounting number of studies have revealed that glutaminolysis plays an indelible role in the differentiation of CD4^+^ T cells. Besides, alterations in the glutaminolysis can also lead to changes in the fate of peripheral CD4^+^ T cells. All of this indicate that the glutaminolysis pathway has excellent potential for interventional regulation of CD4^+^ T cells differentiation. Here, we summarized the process by which glutaminolysis regulates the fate of CD4^+^ T cells during differentiation and further investigated how to reshape abnormal CD4^+^ T cell differentiation by targeting glutaminolysis.

## 1 Introduction

T cells are derived from pluripotent stem cells of bone marrow, where T cell precursors first develop in the bone marrow, then move to the thymus for further programming and development, and gradually differentiate into T cells with immune activity in the thymus (1). These T cells are transported by the bloodstream to the lymph nodes, peripheral blood, and immune tissues where they colonize and take their final organ-specific characteristics (1). These peripheral naive T cells can be divided into two major subsets, CD4^+^ and CD8^+^ T cells, based on the CD cluster (CD) on their surfaces. Naive CD4^+^ T cells can differentiate into a variety of forms following antigen stimulation, including T helper (Th1, Th2, Th9, Th17, and Th22) cells, Treg cells, T memory (Tm) cells, and T follicular helper (Tfh) cells (2–4). In general, CD4^+^ T cells primarily perform the tasks of cytotoxicity, accessory immunity, and immune regulation (5). Each subtype specifically has distinct roles, but the differentiation of Th1, Th17, and Treg cells is of special interest because changes in the balance of these cells have been linked to a variety of illnesses, including autoimmune diseases, transplant rejection, and irritability (6, 7). For example, in systemic lupus erythematosus (SLE), excessive differentiation of Th17 cells and reduced differentiation of Treg cells are the main causes of disease development and tissue damage (8). Besides, Th1 and Th17 cells are closely related to the occurrence of this pathological state in acute cellular rejection induced by organ transplantation (9). Hence, it is crucial to restore the equilibrium of different subtypes of CD4^+^ T cells in diseases and stabilize it in healthy organisms.

Glutamine (Gln), a kind of immune regulatory nutrient, is frequently used in large amounts to supply cellular energy and to supply intracellular synthesis of genetic material *via* glutaminolysis within rapidly proliferating/dividing cells (10). Therefore, the original focus of studies on the effects of glutaminolysis on cells was tumorigenesis. Since immune cells also require substantial proliferation to function after activation, there has been progressively increasing research focusing on glutaminolysis in immune cells in recent years. Immunometabolism mainly investigates the reciprocal influence of immunity and metabolism in physiology and disease, with the ultimate goal of harnessing the distinct metabolic programs of different immune cell populations to treat disease. With the progress of immunometabolism, the role of Gln, considered as an immunomodulatory nutrient, has been gradually unraveled in immune related diseases. For instance, administration of glutaminolysis enzyme inhibitors can increase the acceptance of allografts in a mouse skin transplant model (11). Similar to this, in a mouse psoriasis model, aberrant glutaminolysis activation can cause lesion aggravation by promoting Th17 cell differentiation (12). These studies suggest that glutaminolysis may play a crucial role in immune related diseases and that the generation of these effects seems to be closely related to CD4^+^ T cell differentiation. As a result, manipulating glutaminolysis to reshape CD4^+^ T cell differentiation appears to be an effective intervention for immune-related diseases. In this review, we provide an overview on the role of glutaminolysis in peripheral CD4^+^ T cell differentiation and on the potential points of intervention in the glutaminolysis pathway for the treatment of various diseases.

## 2 Glutaminolysis

**Figure 1:**
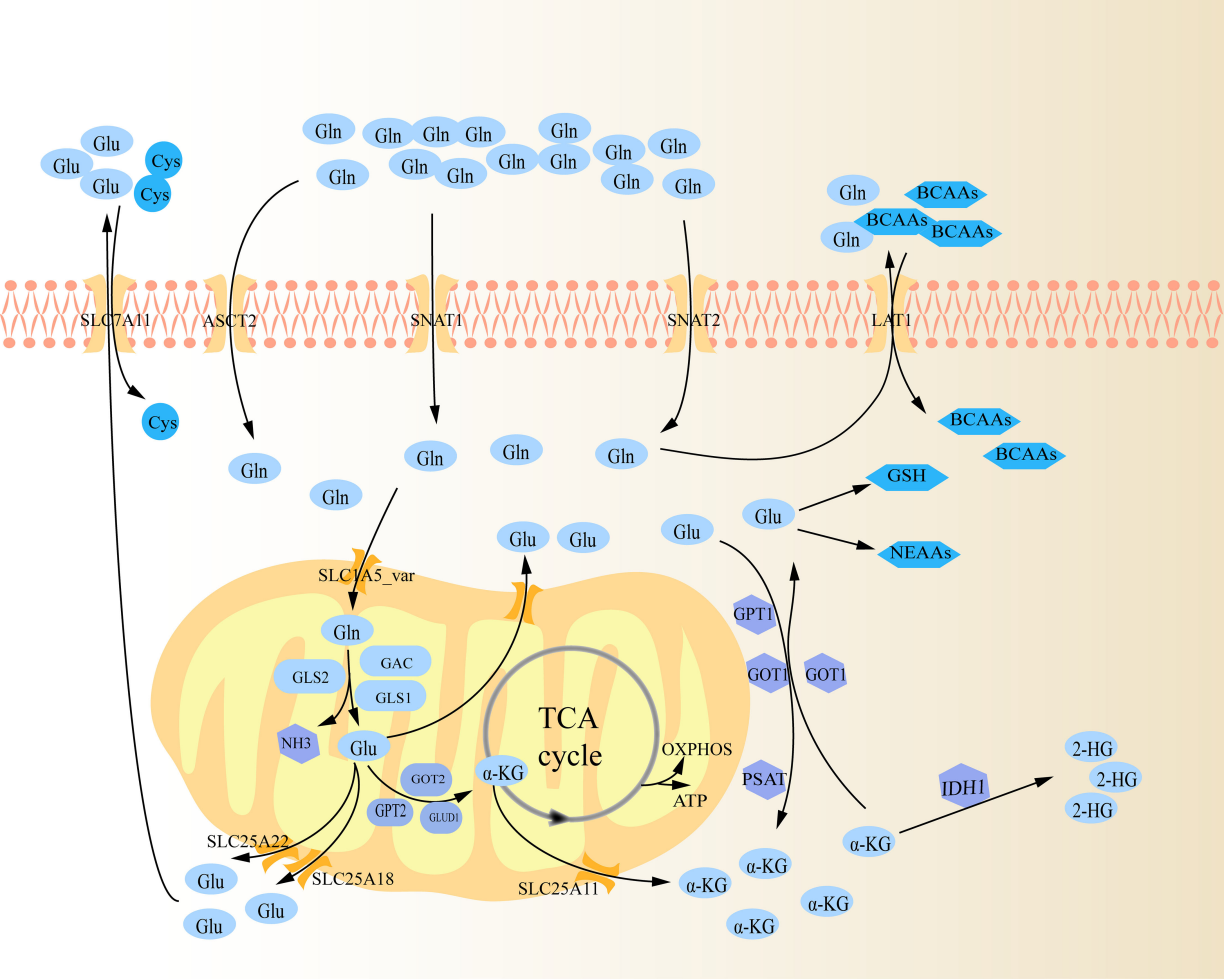
Schematic diagram of glutaminolysis. Gln is ingested into cells through several amino acid transporters (ASCT2, SNAT1, and SNAT2), and further transported into mitochondria through carriers on the mitochondrial membrane (SLC1A5_var). It is gradually decomposed within the mitochondria by various metabolic enzymes, and then the metabolites are transported out of the mitochondria to perform their functions respectively.

Glutaminolysis is the process by which cells convert Gln to tricarboxylic acid (TCA) cycle metabolites through the activity of multiple enzymes (**Figure 1**) (10). To begin with, Gln infiltrates the cytoplasm through amino acid transporters (AATs), which are a type of membrane bound transport proteins that can mediate the transfer of amino acids into and out of cells or organelles. These transporters are mostly sodium ion-dependent neutral AATs, mainly utilizing the concentration gradient of intracellular and extracellular sodium ions to synergistically transport sodium ions and Gln into cells, and then expel excess sodium ions from cells through a sodium ion pump (13). These transporters primarily consist of solute carrier family 1 member 5 (SLC1A5, namely alanine serine and cysteine transporter system 2, ASCT2), SLC38A1 (sodium-coupled neutral amino acid transporters 1, SNAT1) and SLC38A2 (sodium-coupled neutral amino acid transporters 2, SNAT2), with ASCT2 being the most important (14, 15). Subsequently, Gln enters mitochondria *via* SLC1A5 variant (SLC1A5_var), an AAT that locates on the mitochondrial membrane through its N-terminal targeting signal, then is decomposed into glutamate (Glu) and ammonia under the action of mitochondrial glutaminase (GLS), which is also the rate-limiting step of glutaminolysis (14, 16). GLS is the first enzyme in glutaminolysis, mainly including GLS1, GLS2, and GLS1 splicing isomer (Glutaminase C, GAC) (14). On the one hand, in mitochondria, Glu is then transformed into α-Ketoglutaric acid (α-KG) *via* glutamate dehydrogenase 1 (GLUD1), glutamic oxaloacetic transaminase 2 (GOT2) and glutamate pyruvate transaminase 2 (GPT2) (10). Specifically, under the catalysis of these three enzymes, Glu not only produces α-KG, but also produces ammonia, aspartate and alanine, respectively (10, 17). The intramitochondrial α-KG can participate in the TCA cycle, supporting the oxidative phosphorylation (OXPHOS) pathway and ATP generation (14). Glu and α-KG produced in the mitochondria are transported out *via* SLC25A18, SLC25A22 and SLC25A11 on the mitochondrial membrane respectively (14). On the other hand, Glu can also be converted into α-KG in cytoplasm by a group of transaminases, including GOT1, GPT1 and phosphoserine transaminase 1 (PSAT 1) (10). Similarly, GOT1 and GPT1 catalyze Glu to produce aspartate and alanine, respectively (17). PSAT1 is one of the key enzymes in the serine synthesis pathway, and it also produces a portion of α-KG in the pathway of catalyzing serine synthesis (18). Intracellular α-KG can be further catalyzed to generate 2-hydroxyglutarate (2-HG) by isocitrate dehydrogenase (IDH1), a missense mutant metabolizing enzymes (19). In turn, intracytoplasmic α-KG can be regenerated to Glu *via* GOT1 (10). Cytosolic Glu is involved in the biosynthesis of glutathione (GSH) and non-essential amino acids (NEAAs, e.g. alanine, proline, aspartate, asparagine and arginine) (14). Subsequently, Glu is transported out of the cell *via* SLC7A11, exchanging with cystine (Cys) (14). Likewise, excess Gln in the cytoplasm exchanges extracellular branched-chain amino acids (BCAAs, e.g. leucine, valine, and isoleucine) through SLC7A5 (LAT1) (20).

## 3 Mechanism of glutaminolysis in regulating peripheral naïve CD4^+^ T cell differentiation

**Figure 2:**
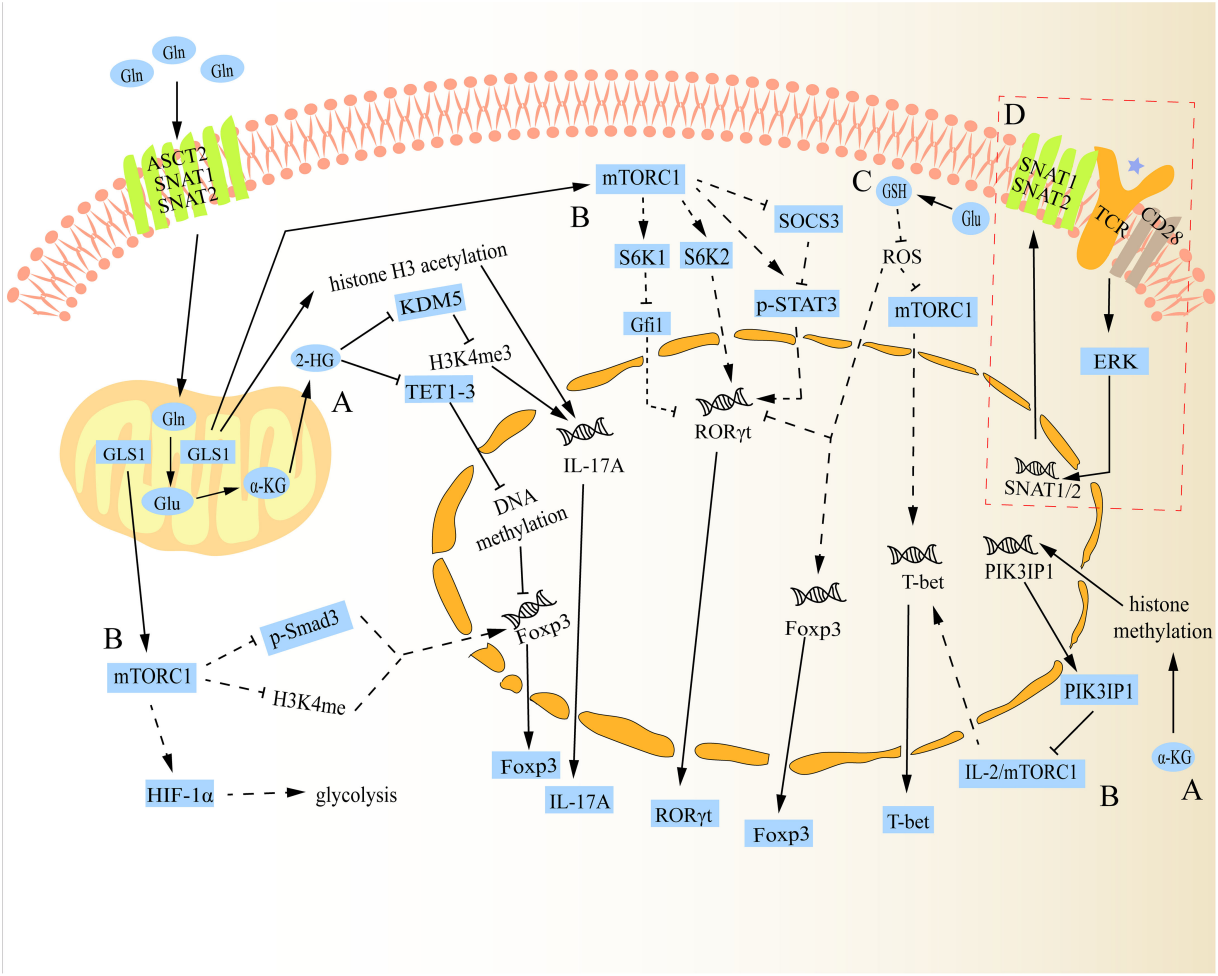
Schematic diagram of the mechanism of glutaminolysis regulating the differentiation of naive CD4^+^ T cells. **(A)** Gln metabolite α-KG can be further catalysed into 2-HG, which can alter the fate of Th17 and Treg cells through regulatory epigenetic regulation. Besides, α-KG can also influence the expression of mTORC1 by epigenetic regulation to change the fate of Th1 cells. **(B)** In addition to regulating the expression of Th1 cells, mTORC1 can also alter the fate of Th17 and Treg cells through various mechanisms. **(C)** Gln metabolite Glu can regulate the differentiation of Th17 and Treg cells *via* Glu-GSH pathway. Additionally, it can also regulate Th1 cell differentiation, but there is a paradox between GSH regulating Th1 cell differentiation and mTORC1 regulating its differentiation. **(D)** After TCR activation, SNAT1/2 expression is up-regulated and promotes its membrane localization. The solid lines in the figure show the direct relationship between glutaminolysis and naive CD4^+^ T cell differentiation reported in studies. The dotted lines in the figure indicate that no study has reported a direct relationship between glutaminolysis and naive CD4^+^ T cell differentiation.

As previously mentioned, peripheral naïve CD4^+^ T cells can differentiate into different subtypes following antigen stimulation. Concretely, the first signal of cell activation is specifically obtained by naive CD4^+^ T cells through the interaction of their T cell receptor (TCR) with the antigenic peptide MHC class II (MHC-II) molecular complex that is displayed on the surface of antigen presenting cells (APCs) (21). Then, the second signal of cell activation is produced when these naive CD4^+^ T cells combine with the corresponding ligand (such as B7) on the surface of APCs and the costimulatory molecule (such as CD28) expressed on its surface (21). In response to dual signals, naïve CD4^+^ T cells become activated, immediately after which they need to take up large amounts of Gln and glucose to meet the biosynthesis materials and energy required for proliferation/differentiation (21, 22). Further investigation revealed that this shift was brought about by an increase in SNAT1, SNAT2, and ASCT2 expression when TCR was activated (23, 24). Naive CD4^+^ T cells subsequently differentiate into various subtypes under the influence of various cytokines in the microenvironment. For example, Th0 cells can polarize into Th1 cells when exposed to cytokines like IL-12, whereas Th0 cells can polarize into Th2 cells when exposed to cytokines like IL-4 (25, 26). Studies have shown that even in specific cell differentiation conditions, impairment in glutaminolysis can have a substantial impact on the fate of peripheral naive CD4^+^ T cells during differentiation (27). The underlying mechanism for how glutaminolysis regulates CD4^+^ T cell differentiation is discussed below (**Figure 2**).

### 3.1 Regulating peripheral CD4^+^ T cell differentiation *via* epigenetic regulation

As a metabolite of intracellular glutaminolysis, α-KG not only participates in the TCA cycle and the generation of other amino acids, but also participates in the regulation of histone and DNA methylation levels as a cofactor of peroxidase, thus participating in the regulation of gene expression in cells (19, 28). Research revealed that Th17 cells produced more α-KG than Treg cells, suggesting that glutaminolysis may be more active in Th17 cells (29, 30). With studies advancing, 2-HG, the actual molecules behind the role of α-KG, was identified. Xu et al. found that under the condition of Th17 cells, cells can penetrate 2-HG, not α-KG, up-regulate the expression of IL-17A, and down-regulate the expression of Foxp3 in a dose-dependent manner, directly promoting the differentiation of Th17 cells (30). Surprisingly, the addition of 2-HG to naïve CD4^+^ T cells even inhibited the expression of Foxp3 under the condition of Treg cells (30), suggesting that 2-HG has obvious differentiation regulation. A subsequent study reported that 2-HG can trigger the DNA methylation of Foxp3 to inhibit its transcription, thus suppressing the differentiation of Treg cells and regulating the homeostasis of Th17/Treg cells (30). Mechanistically, this effect depends on the negative regulation of 2-HG on Tet Methylcytosine Dioxygenase 1-3 (TET1-3), a negative regulator of DNA methylation (30, 31). Besides, Miao et al. verified that 2-HG can facilitate the differentiation of Th17 cells by forming H3K4me3 (Histone H3, trimethylated lysine 4) modifications in the promoter and CNS2 region of the IL-17A gene locus. This effect is made possible by inhibiting KDM5, a lysine demethylase (32). Another independent study showed that GLS1-mediated glutaminolysis was abnormally activated in psoriasis patients and mouse models, which promoted Th17 cell differentiation by enhancing histone H3 acetylation of IL-17A promoter (12). The function of α-KG or 2-HG in the acetylation of H3 histone, however, was not further investigated in their experiments. As for Th1 cells, another type of inflammatory cells, it was shown that Gln deprivation inhibits the differentiation of naïve CD4^+^ T cells into Th1 cells and increases the generation of Foxp3^+^ Treg cells, and this effect can be reversed by the α-KG analogue (33), indicating that it also plays a role in the differentiation of Th1 cells. In line with this, Nakaya et al. found that ASCT2 deficiency hinders Th1 and Th17 cell differentiation, reducing inflammatory T cell responses in a mouse autoimmune model (24). Interestingly, one study claimed that the transient inhibition of GLS1 resulted in an increase in the number of Th1 cells, but it would be exhausted over time (34). In details, after administration of GLS1 blockade, the reduced level of histone methylation in naïve CD4^+^ T cells led to the reduced expression of PIK3IP1,a negative regulator of mTORC1, further leading to the activation of mTORC1 to promote the differentiation of effector Th1 cells (34). Besides, the inhibition of GLS1 also leads to histone modification, thereby increasing the expression level of IL-2, which is more conducive to Th1 differentiation (34, 35). However, only GLS1 inhibition may cause a reduction in intracellular Glu and an accumulation of Gln. In contrast, an excessive amount of Gln accumulation may have the reverse effect. In order to investigate this distinction, more research should be done in the future on the direction and function of intracellular Gln after GLS1 inhibition.

### 3.2 Regulating peripheral CD4^+^ T cell differentiation *via* mTORC1

The activity of mTORC1 plays an important role in integrating the metabolic spectrum and guiding the fate decision of CD4^+^ T cells because it senses and integrates multiple signals from the environment to control metabolism (36). Previous research had demonstrated that Rheb (the positive regulatory target of mTORC1) deficient CD4^+^ T cells suppressed the differentiation of Th1 cells by reducing the response to IL-12 and preventing T-bet transcription (37, 38). On the contrary, Rheb deficient CD4^+^ T cells showed enhanced phosphorylated STAT6 level in response to IL-4 (Th2 cell polarization factor), which further increased the transcription level of GATA3 in cell nuclear, thus promoting the differentiation of Th2 cells (39). Regarding Th17 cells, to begin with, the activation of mTORC1 leads to an increase in STAT3 phosphorylation at tyrosine 705 in naive CD4^+^ T cells, which is required for the RORγt genes expression (39). Then, mTORC1 promotes glycolysis by inducing HIF-1α, which in turn supports the differentiation of Th17 cells (40). Besides, mTORC1 enhances the differentiation of Th17 cells in a way that is dependent on S6K1/2, where S6K1 inhibits the down-regulation of Gfi1, a negative regulator of Th17 cell differentiation, and S6K2 enhances the nuclear localization of RORγt (41). Lastly, by blocking SOCS3 (a negative regulator of STAT3), mTORC1 can also promote STAT3 phosphorylation and RORγt expression that are induced by IL-6 (39). However, the function of mTORC1 is reversed during the differentiation of Treg cells. One example is that mTORC1 blocks the development of Treg cells by preventing Smad3 phosphorylation or H3K4 methylation close to the Foxp3 transcription start site, both of which have been shown to encourage Foxp3 transcription (41). Another example is that mTORC1 can increase glycolytic activity *via* inducing HIF-1α, but Treg cells is less dependent on glycolytic metabolic procedure to provide energy compared with Th17 cells, thus leading to a significant difference in the differentiation of Th17 and Treg cells (40, 42).

Amino acids play an important role in the activation of mTORC1 signaling pathway, especially Gln, leucine (Leu), arginine (Arg) and methionine (Met) (24, 43–45). ASCT2, an essential amino acids transporter, is mainly responsible for Gln transporting into cells (46). Additionally, it is in charge of bringing a tiny quantity of Leu into cells (24). ASCT2 has also been identified as necessary for coupling TCR and CD28 signals to activate the mTORC1 pathway (47). Nakaya et al. revealed that a lack of ASCT2 in naive CD4^+^ T cells reduced the differentiation of Th1 and Th17 cells by attenuating the uptake of Gln and Leu to suppress mTORC1 activation (24). However, they were unable to identify which amino acid (Gln or Leu) intake was reduced as a result of the inhibition of mTORC1 activity brought on by ASCT2 knockout. Recently, Zhang et al. showed that blocking GLS1 promoted Th2 cell differentiation and inhibited Th17 cell differentiation through inactivating the mTORC1 pathway, but they did not notice any changes in Th1 cell differentiation (48). Similar finding was made by Nakaya et al. who discovered that reducing Gln consumption by eliminating ASCT2 could enhance Th2 cell differentiation (24). Taken together, even though the impact of Leu on the activation of mTORC1 has not been completely ruled out in recent studies, glutaminolysis does play a significant role in the differentiation of naive CD4^+^ T cells by regulating mTORC1.

### 3.3 Regulating peripheral CD4^+^ T cell differentiation *via* GSH

Under physiological conditions, glutathione exists mainly in two forms, reduced glutathione (GSH) and oxidized glutathione (GSSG), which can interconvert (49). Intracellular GSH is mainly produced by two pathways, *de novo* synthesis (via glutaminolysis) and recycling process (via the regeneration of GSH from GSSG) (49). The primary cellular antioxidant, GSH, which is primarily made up of Glu, Cys, and glycine (Gly), is responsible for preserving the redox balance in T cells (50). According to a prior research, increasing ROS by inhibiting GSH *de novo* synthesis but not recycling increased intracellular GSH production, which ultimately improved Treg cell differentiation and restricted Th17 cell differentiation (51). Also, they proved that glutaminolysis is the source of Glu, which powers *de novo* GSH production during the differentiation of Th17 cells (51). Furthermore, Miao et al. discovered that inhibiting GLS1-mediated glutaminolysis decreased intracellular GSH, which raised ROS levels to suppress RORγt expression, the key transcription factor for Th17 cell development (32). Subsequent mechanism research revealed that GSH produced from *de novo* synthesis buffers ROS to relieve its inhibition on mTORC1, inducing Th17 cell differentiation (52). As for Th1 cells, studies had shown that administration of GSH supplementation promoted Th1 cell differentiation at the time of viral invasion (53, 54). It has been reported that high levels of GSH can cause APCs to release more IL-12, which can help Th1 cells to differentiate (25). On the contrary, the consumption of GSH led to the decrease of IL-12 secretion, induced the production of IL-4, inhibited the production of Th1-related cytokines and/or promotes Th2-related reactions (55). Nevertheless, the detailed regulatory mechanisms by which GSH regulates Th1/Th2 cells differentiation are still unclear.

## 4 Intervention strategy to harness glutaminolysis for immunotherapy

The homeostasis of CD4^+^ T cells is particularly important for the maintenance of organismal health, as several diseases have been linked to aberrant CD4^+^ T cell differentiation. For example, when Th1 and Th17 cells are differentiated excessively while Treg cell differentiation is insufficient, hyper immunological illnesses such as autoimmune diseases, graft rejection, and irritability result (56, 57). Hence, reshaping the disordered CD4^+^ T cell subsets is a simple and promising way to achieve immunotherapy. Specifically, intervention is required in humans to prevent the differentiation of peripheral naïve CD4^+^ T cells into proinflammatory cells in hyper immunological disorders. As previously described, the glutaminolysis pathway is a proper site of intervention to regulate the differentiation of naïve CD4^+^ T cells. In a nutshell, glutaminolysis is primarily separated into two steps: intracellular Gln uptake and progressive degradation. Therefore, from these features, intervention strategies to harness glutaminolysis for immunotherapy can also be developed (see **Table 1** for details).

**Table 1:** Regulatory interventional strategies for glutaminolysis.

Strategy	Target	Regulator/Drug	Mechanism	Ref
Interference with Gln uptake	ASCT2	RNF5	Down-regulating ASCT2 expression by mediating ASCT2 ubiquitination	(58)
		Leptin	Inhibiting ASCT2 function by inhibiting Na ion flow	(59)
		Insulin	Up-regulating ASCT2 expression by activating ERK cascade	(60)
		MiR-137	Down-regulating ASCT2 expression by sponging with its mRNA	(61, 62)
		Benzyl-serine/cysteine/glycine, GPNA	Competitively inhibiting ASCT2 as Gln analog	(63–66)
		1,2,3-dithiazoles	Inhibiting ASCT2 function by forming mixed sulfide with Cys residue of protein	(67)
		TPT, RV, δT	Unknown	(68–70)
		Ab3-8, KM4008, KM4012, KM4018 and KM8094 mAbs	Inhibiting ASCT2 function though targeting cell surface domains of ASCT2	(71–73)
	SNAT1/2	ERK	Up-regulating SNAT1 and SNAT2 expression by activating ERK cascade	(23)
		GPNA	Competitively inhibiting SNAT1 and SNAT2 as Gln analog	(15, 74)
		MeAIB	Competitively inhibiting SNAT1 and SNAT2 as Gln analog	(75)
	SNAT2	Compound 12, V-9302	Unknown	(76)
	CBM complex	CARMA1	Down-regulating CARMA1 expression by its ubiquitination and phosphorylation	(77–79)
		BCL10	Down-regulating BCL10 expression by its ubiquitination and phosphorylation	(79, 80)
		MALT1	Down-regulating MALT1 expression by its ubiquitination and phosphorylation	(79)
		MALT1 inhibitor	Inhibiting CBM complex function by blocking MALT1	(81)
Interference with Gln enzymolysis	GLS1	MiR-145, miR-23a/b, miR-194 and miR-204	Inhibiting GLS1 expression through sponging 3’-UTR of GLS1 mRNA	(82–84)
		PPARγ	Down-regulating GLS1 gene expression by forming heterodimers with retinoid X receptor	(32, 85, 86)
		ICER	Enhancing its activity by binding to the GLS1 promoter directly	(29)
Interference with Gln enzymolysis	GLS1	HIF-1α	Up-regulating GLS1 expression *via* binding to hypoxia-responsive element in the gene	(85)
		DON	Inhibiting GLS1 activity through covalent modification of the ser286 site as Gln analog	(86)
		BPTES, apomorphine	Inhibiting GLS1 activity by stabilizing the inactive tetramer	(87, 88)
		compound 968, CB-839	Allosteric inhibitors of GLS1	(89, 90)
		ebselen	Inhibiting GLS1 activity by forming a selenyl sulfide (–Se–S–) bond with the cys residue of proteins	(88)
		chelerythrine	Inhibiting GLS1 activity by covalent modification of its imine moiety and the thiol group on proteins	(88)
	GOT1	AOA	Unknown	(30)
	GAC	BPTES	Inhibiting GAC activity by stabilizing the inactive tetramer	(87)
		compound 968, CB-839, compound 19, UPGL00004	Allosteric inhibitors of GAC	(89–92)
Simultaneous interference with Gln uptake and enzymolysis	ASCT2, GLS1	Rb	Down-regulating mRNA transcription of ASCT2 by inhibiting transcription factor E2F3; Directly inhibiting GLS1 expression	(93)
	ASCT2, SNAT1/2, GLS1	c-Myc	Proteomic finding; Up-regulating target gene expression by acting as a transcription factor probably	(94)

### 4.1 Interference with Gln uptake

#### 4.1.1 ASCT2

ASCT2 is a homotrimer encoded by SLC1A5 gene, which is the main amino acid carrier for Gln transport into cells (95). Previous studies had revealed that inhibiting Gln uptake *via* targeting ASCT2 could lead to decreased differentiation of Th1 and Th17 cells meanwhile increased differentiation of Treg cells (24, 33). As a result, ASCT2 is a good candidate for intervention. Although the regulatory mechanism of ASCT2 is still unclear, the following methods have been described how to regulate ASCT2 *in vivo*. RNF5, a kind of E3 ubiquitin-protein ligase, can mediate the ubiquitination of ASCT2, leading to the down-regulated expression of ASCT2 (58). White adipocytes secrete a protein called leptin into the bloodstream, which is important for controlling energy homeostasis and can prevent Gln uptake by suppressing ASCT2 expression (59). In addition, it has been noted that insulin activates the ERK cascade to promote ASCT2-mediated Gln transport (60), suggesting that insulin antagonists may have some effect on ASCT2 inhibition. A potent class of non-coding RNAs known as microRNAs (miRNAs) controls gene expression by interacting with target mRNAs to either prevent their translation or promote their destruction (96). Studies have shown that miR-137 can bind to the mRNA of ASCT2, which in turn down-regulates the expression of ASCT2, inhibiting the Gln uptake (61, 62).

Currently, research on the pharmacological intervention of ASCT2 is a focus in addition to the regulatory targets of ASCT2. Benzyl-serine, benzyl-cysteine, and phenyl-glycine have all been reported to inhibit ASCT2 competitively, but they are not specific ASCT2 inhibitors because they also block the other transporters such as LAT1 and ASCT1 (63–65). L-γ-glutamyl-p-nitroanilide (GPNA), an analog of Gln, is a kind of non-specifical blockade of ASCT2 (66). Except for ASCT2 blockade, GPNA can also block SNAT1, SNAT2 and LAT1 (15, 74). A recent study showed that in a mouse asthma model based on ovalbumin, the administration of GPNA significantly alleviated the asthma state and reduced the level of inflammatory cells infiltration in the body (97). The thiol/thiolate groups of Cys are involved in covalent interactions with 1,2,3-dithiazoles, which in turn impede ASCT2 function (67). Besides, topotecan (TPT), resveratrol (RV) and δ-tocotrienol (δT) have also been reported to inhibit ASCT2 (68–70). Monoclonal antibody (mAb) development is another area of study. Studies have demonstrated that the mAbs Ab3-8, KM4008, KM4012, KM4018, and KM8094 can reduce Gln uptake by focusing on ASCT2’s cell surface domains (71–73). In a word, since the majority of the currently available ASCT2 inhibitors are non-specific, which may contribute to inhibition of some other AATs, leading to deficiencies in the uptake of some other amino acids, new ASCT2 inhibitory medications must be developed.

#### 4.1.2 SNAT1 and SNAT2

Targeting SNAT1 and SNAT2, encoded by SLC38A1 and SLC38A2 respectively, is the other intervention technique to reduce Gln uptake into naïve CD4^+^ T cells because they are both capable of mediating Gln transport into cells (98). Even though the regulation mechanisms of SNAT proteins are currently less explored, the following processes also show some precedent significance. Previous research had shown that downstream ERK activation was enhanced after TCR activation, further resulting in the up-regulation of SNAT1 and SNAT2 to promote Gln uptake (23). Hence, targeting ERK cascades seems to be a promising intervention point to achieve the regulation of SNAT1 and SNAT2. Moreover, pharmacological inhibitors, such as GPNA, are also a direction of development. As previously mentioned, GPNA has the ability to non-specifically inhibit SNAT1 and SNAT2 (15, 74). Since SNAT1 and SNAT2 are believed to belong specifically to amino acid transport system A (ATA), N-methyl-aminoisobutyric acid (MeAIB), a substrate of ATA, can bind competitively with Gln to inhibit SNAT1 and SNAT2 (75, 98). Initially thought to be a competitive inhibitor of ASCT2, compound 12 and its isomer V-9302, derived from 2-amino-4-bis(aryloxybenzyl)aminobutanoic acids, were later identified as an inhibitor of SNAT2 as research advanced (76). Combined with the present study, the study of regulation of SNAT proteins is still lacking, and therefore, it will be a potential direction of research.

#### 4.1.3 CBM complex

The CBM complex is composed of the scaffolding protein CARMA1, the adaptor protein BCL10, and the para-caspase enzyme MALT1 (99). It was previously shown that the CBM complex acted as a bridge to transmit the activated TCR signal to the downstream IKK/NF-κB and c-Jun N-terminal kinase (JNK) pathways, thereby causing T cell activation (100). Nakaya et al. found that knockout of any one of the constituent proteins of the CBM complex can result in attenuated Gln uptake and in particular loss of CARMA1 also resulted in down-regulation of ASCT2 mRNA levels both basal and after TCR stimulation (24). As a result, by assisting Gln to enter cells during signal transduction after TCR stimulation, the CBM complex functions more as an intermediary bridge. The primary regulatory mechanisms for the CBM complex, according to the available reports, are its ubiquitination and phosphorylation (77). For example, CARMA1 may be ubiquitinated by the E3 ubiquitin-protein ligase CBL-b, which will subsequently cause it to be degraded (78). There are several CARMA1-related regulatory mechanisms of phosphorylation and ubiquitination, aside from the CBL-b, that have been well examined (for more information, see refs (77, 79)). Similar to CARMA1, these two methods equally regulate BCL10 and MALT1. For instance, the NEMO/IKKβ complex has the ability to phosphorylate BCL10 at Thr-81 and Ser-85, leading to BCL10 destruction through the lysosomal pathway (80). More relevant regulatory mechanisms about the ubiquitination and phosphorylation of CBM complex can be found in ref (79). Contrary to CARMA1 and BCL10, MALT1 has a strong foundation in pharmaceutical research because it is the only human para-caspase that has received significant attention as an immunomodulatory target for the treatment of autoimmune and inflammatory illnesses. To disturb MALT1, numerous compounds have been created, and drug clinical trials have even started (see (81) for more information). Overall, greater research in this area is worthwhile because the CBM complex appears to be a novel and promising intervention target for reducing the uptake of Gln.

### 4.2 Interference with Gln enzymolysis

#### 4.2.1 GLS1

GLS1, located in mitochondria, is the first enzyme of glutaminolysis, which plays a role in regulating cell metabolism, maintaining cell redox balance and GSH biosynthesis (14). Previous research has demonstrated that blocking GLS1 could result in increased Treg cell differentiation and decreased Th17 cell differentiation (12, 32). Thus, in order to restore the balance of Th17/Treg cells in some disorders brought on by Th17 over-differentiation, targeting GLS1 may be a promising approach. GLS1 is a type of enzyme that can be regulated *in vivo* through a variety of ways. For example, by sponging the 3’-UTR of GLS1 mRNA, miR-145 and miR-23a/b can decrease the expression of GLS1 (82, 83). In addition, database mining research revealed that miR-194 and miR-204 might specifically target GLS1 and limit its expression (84). By forming heterodimers with the retinoid X receptor (RXR), the transcription factor peroxisome proliferator-activated receptor gamma (PPARγ) can regulate the expression of its target genes (101). Miao et al. revealed that PPARγ agonists could remold the balance of Th17/Treg cells *via* down-regulate GLS1 expression in dextran sulfate sodium (DSS)-induced colitis and house dust mite (HDM)/lipopolysaccharide (LPS)-induced asthma mouse models (32). Consistently, Yang et al. used Bergenin, a PPARγ agonist, to block the differentiation of naive CD4^+^ T cells into Th17 cells by inhibiting GLS1-dependent glutaminolysis under Th17-polarizing condition, thus alleviating asthma in mouse model (102). The transcription factor inducible cAMP early repressor (ICER) had been shown to enhance its activity by binding to the GLS1 promoter directly, promoting the differentiation of Th17 cells (29). HIF-1α can also increase the expression of GLS1 *via* binding to the hypoxia-responsive element (HRE) in the GLS1 gene (85). Thus, inhibiting ICER and HIF-1α by exploring methods is also a reliable strategy for GLS1 blockade. Besides, a series of inhibitors have been developed to target GLS1. 6-diazo-5-oxo-L-norleucine (DON), a common GLS1 inhibitor, competitively inhibits GLS1 by acting as a substrate Gln analogue (86). Specifically, DON binds to the active site of GLS1 by covalently modifying the ser286 site, preventing GLS1 from functioning (86). Bis-2-(5-phenylacetamido-1,3,4-thiadiazol-2-yl) ethyl sulfide (BPTES) is a GLS1 non-selective inhibitor, which can function by stabilizing the inactive tetramer (87). According to research, administering the GLS1 inhibitor BPTES reduced the excessive differentiation of Th17 cells in naïve CD4^+^ T cells from SLE patients, which is consistent with the results of GLS1 conditional knockout in the experimental autoimmune encephalomyelitis mouse model (103). Besides, 5-[3-Bromo-4-(dimethylamino) phenyl]-2, 3, 5, 6-tetrahydro-2-dimethyl-benzo [a] phenanth-ridin-4 (1H)-one (namely compound 968), telaglenastat (CB-839), ebselen, chelerythrine and apomorphine are also GLS1 inhibitor reportedly (88–90). An example is that after intraperitoneal injection of GLS1 CB-839, the imbalance of Th1/Th2 and Th17/Treg was rectified, alleviating the SLE development (48).

#### 4.2.2 Other enzymes

Except for GLS1, GAC, GLUD1, GOT1, GOT2, GPT2 and IDH1 are other metabolic enzymes of glutaminolysis, implying that they are also potential regulatory targets. For instance, by reshaping the balance between Th17 and Treg cells, selective inhibition of GOT1 with (aminooxy)acetic acid (AOA) ameliorates experimental autoimmune encephalomyelitis in mice (30). Several GLS1 inhibitors (BPTES, compound 968, CB-839), compound 19 and UPGL00004 have also been reported to inhibit GAC (87, 89–92). Besides, IDH1, the primary catalytic enzyme for 2-HG synthesis and a key regulator of naive CD4^+^ T cell differentiation, has enormous promise as a target for therapeutic intervention. Therefore, more mechanistic research is required to establish the foundation for future target discovery for these downstream metabolic enzymes.

Nevertheless, current studies on the inhibition of Gln metabolizing enzymes mainly focus on Th17 and Treg cells. Inhibition of glutaminolysis enzymes GLS1 alone may cause aberrant Th1 cell differentiation, as revealed by Johnson (34). The reason might be related to the inhibition of GLS1, leading to excessive intracellular Gln accumulation, which might in turn undergo some substance exchange and biological reactions *via* certain amino acid transporters, but regretfully, they could not further design to support this theory. Thus, inhibiting Gln metabolizing enzymes alone may be a good treatment in diseases brought on by abnormalities of Th17/Treg cells, but it still requires additional research in conditions where Th1 cells are predominate.

### 4.3 Simultaneous interference with Gln uptake and enzymolysis

Both glutamine uptake and metabolic catabolic enzymes can be regulated simultaneously *in vivo* in addition to being targeted separately. According to reports, the RB transcriptional corepressor (Rb) can adversely regulate both ASCT2 and GLS1 expression at the same time. Deletion of Rb can both increase ASCT2 mRNA transcription through an E2F3-dependent mechanism and directly suppress GLS1 expression (93). The function of the c-Myc protein in T cells, which functions as a genetic switch to regulate a number of cellular metabolisms, has been discovered in recent years. Studies had revealed that upon TCR activation, metabolic reprograming of T cells occurred *via* up-regulating c-Myc (94, 104). Moreover, it is found that up-regulated c-Myc in T cells could cause the up-regulated expression of ASCT2, SNAT1, SNAT2 and LAT1 by proteomic analysis (94). Therefore, these data suggest that simultaneous inhibition of Gln uptake and enzymolysis can be achieved by inhibiting c-Myc. Previous studies had shown that immune checkpoints such as CTLA-4, PD-L1 in tumor cell could prevent TCR activation and, therefore further inhibiting downstream metabolic reprogramming (105). In this regard, designing to up-regulate these expressions in immune-excessive non-neoplastic diseases is perhaps also a therapeutic strategy *via* blocking the Gln uptake and enzymolysis in T cells.

## 5 Conclusion

In conclusion, glutaminolysis plays an irreplaceable role during the differentiation of peripheral CD4^+^ T cells. Upon TCR activation, naïve CD4^+^ T cells start taking up Gln in large amounts to promote Th1, Th17 cell differentiation and inhibit Treg cell differentiation through several mechanisms including epigenetic regulation, mTORC1 activation and GSH pathway. After inhibition of glutaminolysis, there was an opposite trend in peripheral naïve CD4^+^ T cell differentiation. Therefore, regulation of peripheral Th1, Th17, and Treg cell differentiation by intervening glutaminolysis in naïve CD4^+^ T cells shows great potential to be exploited in immune-excessive diseases. Besides, in other diseases such as tumor, as an infinitely proliferating cell, it requires a large amount of Gln uptake and decomposition, as do anti-tumor CD4^+^ T cells such as Th1 and Th17 cells. So, although inhibition of Glutaminolysis in tumor cells is effective, it has not completely cleared the tumor lesions, which may be related to the tendency of CD4^+^ T differentiation changes to Treg after Glutaminolysis inhibition. Meanwhile, although there are many studies focusing on glutaminolysis and naïve CD4^+^ T cell differentiation, most of these studies focused on mouse cells *in vitro*. Studies on human naïve CD4^+^ T cell differentiation are still relatively lacking, and this is perhaps an area in which we deserve to explore in-depth. Moreover, at present, there are a few related reports on the regulation of Th2 cell differentiation by glutaminolysis, and how it regulates Th2 cell differentiation is still unknown, thus it is necessary to carry out related researches. Taken together, we summarized the existing studies, concluding several different interventional strategies for glutaminolysis. These strategies are currently mostly used in tumor diseases, and their application in inflammatory diseases still needs to be experimentally confirmed. Therefore, we expect to be able to guide directions on how to appropriately utilize glutaminolysis for future basic research and clinical applications in inflammatory diseases.

## Author contributions

TL, SR, CS drafted manuscript, have contributed equally to this work and should be considered co-first authors. PZ helped in literature searching. HW provided administrative and financial support, manuscript revision and final approval of the manuscript. All authors contributed to the article and approved the submitted version.
